# The Effect of 3 Versus 6 Years of Zoledronic Acid Treatment of Osteoporosis: A Randomized Extension to the HORIZON-Pivotal Fracture Trial (PFT)

**DOI:** 10.1002/jbmr.1494

**Published:** 2011-12-08

**Authors:** Dennis M Black, Ian R Reid, Steven Boonen, Christina Bucci-Rechtweg, Jane A Cauley, Felicia Cosman, Steven R Cummings, Trisha F Hue, Kurt Lippuner, Peter Lakatos, Ping Chung Leung, Zulema Man, Ruvie Lou Maria Martinez, Monique Tan, Mary Ellen Ruzycky, Guoqin Su, Richard Eastell

**Affiliations:** 1 University of CaliforniaSan Francisco, CA, USA; 2 University of AucklandAuckland, New Zealand; 3 Katholieke Universiteit LeuvenLeuven, Belgium; 4 Novartis Pharmaceuticals CorporationEast Hanover, NJ, USA; 5 University of PittsburghPittsburgh, PA, USA; 6 Helen Hayes HospitalWest Haverstraw, NY, USA; 7 California Pacific Medical CenterSan Francisco, CA, USA; 8 Bern University HospitalBern, Switzerland; 9 Semmelweis University Medical SchoolBudapest, Hungary; 10 Chinese University of Hong Kong, Prince of Wales HospitalHong Kong, China; 11 Centro Médico TIEMPOBuenos Aires, Argentina; 12 University of SheffieldSheffield, UK

**Keywords:** FRACTURE, POSTMENOPAUSAL OSTEOPOROSIS, ZOLEDRONIC ACID, BISPHOSPHONATES, EXTENSION STUDY

## Abstract

Zoledronic acid 5 mg (ZOL) annually for 3 years reduces fracture risk in postmenopausal women with osteoporosis. To investigate long-term effects of ZOL on bone mineral density (BMD) and fracture risk, the Health Outcomes and Reduced Incidence with Zoledronic acid Once Yearly–Pivotal Fracture Trial (HORIZON-PFT) was extended to 6 years. In this international, multicenter, double-blind, placebo-controlled extension trial, 1233 postmenopausal women who received ZOL for 3 years in the core study were randomized to 3 additional years of ZOL (Z6, *n* = 616) or placebo (Z3P3, *n* = 617). The primary endpoint was femoral neck (FN) BMD percentage change from year 3 to 6 in the intent-to-treat (ITT) population. Secondary endpoints included other BMD sites, fractures, biochemical bone turnover markers, and safety. In years 3 to 6, FN-BMD remained constant in Z6 and dropped slightly in Z3P3 (between-treatment difference = 1.04%; 95% confidence interval 0.4 to 1.7; *p* = 0.0009) but remained above pretreatment levels. Other BMD sites showed similar differences. Biochemical markers remained constant in Z6 but rose slightly in Z3P3, remaining well below pretreatment levels in both. New morphometric vertebral fractures were lower in the Z6 (*n* = 14) versus Z3P3 (*n* = 30) group (odds ratio = 0.51; *p* = 0.035), whereas other fractures were not different. Significantly more Z6 patients had a transient increase in serum creatinine >0.5 mg/dL (0.65% versus 2.94% in Z3P3). Nonsignificant increases in Z6 of atrial fibrillation serious adverse events (2.0% versus 1.1% in Z3P3; *p* = 0.26) and stroke (3.1% versus 1.5% in Z3P3; *p* = 0.06) were seen. Postdose symptoms were similar in both groups. Reports of hypertension were significantly lower in Z6 versus Z3P3 (7.8% versus 15.1%, *p* < 0.001). Small differences in bone density and markers in those who continued versus those who stopped treatment suggest residual effects, and therefore, after 3 years of annual ZOL, many patients may discontinue therapy up to 3 years. However, vertebral fracture reductions suggest that those at high fracture risk, particularly vertebral fracture, may benefit by continued treatment. (ClinicalTrials.gov identifier: NCT00145327). © 2012 American Society for Bone and Mineral Research.

## Introduction

In the Health Outcomes and Reduced Incidence with Zoledronic acid Once Yearly–Pivotal Fracture Trial (HORIZON-PFT), 5 mg zoledronic acid (ZOL), given intravenously annually for 3 years, was shown to decrease spine, hip, and other nonvertebral fracture risk, to increase bone mineral density (BMD), and to decrease bone remodeling rates.^1^ These results, together with those from trials of similar duration for oral bisphosphonates,^2^–^6^ support fracture risk reduction with 3 to 4 years of bisphosphonate administration, particularly in osteoporotic women. However, bisphosphonate efficacy over longer periods has been less well studied. There have been no long-term placebo-controlled trials, but one alendronate study randomized women after 5 years on treatment to either 5 more years of alendronate or placebo.^7^ This study found that those randomized to continue alendronate retained BMD gains, whereas those switched to placebo lost BMD but remained at or above pretreatment levels from 10 years earlier. Clinical vertebral, but not nonvertebral, fractures were reduced among those continuing. The authors recommended that many women could take a “drug holiday” after 5 years but that those at high risk of vertebral fractures might continue. A later post hoc analysis suggested nonvertebral fracture benefits in those with BMD *T*-scores lower than −2.5 after 5 years of treatment.^8^ This long persistence of effect is not true for all bisphosphonates; for example, 1 year after stopping risedronate, there was no difference between the active and placebo groups in bone turnover markers (although BMD was still higher and fracture incidence remained reduced in the active group).^9^

Although 3- to 4-year trials of bisphosphonates have not identified any consistent safety concerns, several safety concerns have arisen from sources other than trials, including osteonecrosis of the jaw (ONJ),^10^ esophageal cancer, and more recently, atypical femur fractures.^11^–^13^ The latter association was not supported by a reanalysis of randomized trials.^14^ However, recent large epidemiologic studies have been more supportive of an association.^11^, ^15^–^17^ Although there is still significant uncertainty about the relationship between bisphosphonate use and duration of use and the risk of atypical fracture, it persists as a safety concern, particularly with long-term bisphosphonate therapy. A Food and Drug Administration (FDA) advisory committee recently recommended that the FDA make some changes to bisphosphonate labels regarding long-term use.^18^

To assess the effect of ZOL beyond 3 years, we conducted an extension of the HORIZON-PFT in which women on ZOL for 3 years were randomly assigned to ZOL or placebo for 3 more years. Our goals were to assess the efficacy and safety of 6 years of ZOL versus 3 years followed by cessation, and to estimate the effect of offset after discontinuation.

## Materials and Methods

### Study design and participants

This trial was an extension of the HORIZON-PFT; the design and results from that core study have previously been reported.^1^ In summary, 7765 osteoporotic women were randomly assigned to annual intravenous ZOL 5 mg or placebo and followed for 3 years. In this extension, women who had received three ZOL or placebo infusions in the core study at a subset of clinical sites were eligible. Exclusions included major protocol violations during the core study, aged >93 years, and specific bone-active medication use. All patients provided written informed consent before participating in the study, and local Independent Ethics Committees or Institutional Review Boards for each participating study center approved the protocol. The study was conducted in compliance with the ethical principles of the Declaration of Helsinki (2008) and local applicable laws and regulations.

The study was jointly designed by the steering committee and sponsor. The sponsor had responsibility for data collection and quality control. An independent data and safety monitoring board (DSMB) met semiannually to oversee study conduct and monitor patient safety. Study database copies were periodically transferred to the University of California, San Francisco (UCSF) for DSMB reports. Analyses for publication were the joint responsibilities of the sponsor and UCSF investigators. Original analyses were performed by the sponsor according to a prespecified analysis plan and independently confirmed by UCSF. All authors contributed to the manuscript, and approval was received from the 13-member Steering Committee, which included two representatives of the sponsor.

### Treatment

Women assigned to ZOL in the core study were randomly assigned to receive a 15-minute intravenous infusion of ZOL (Group Z6) or placebo (Group Z3P3) once per year for 3 years, in a 1:1 ratio, stratified by clinical center. To maintain blinding, patients assigned to placebo during the core study received ZOL in the extension study for 2 to 3 years but will not be considered further in this report. To further ensure blinding, patients were randomized centrally by an interactive voice response system to study treatment. All patients received daily oral calcium (1000 to 1500 mg) and vitamin D (400 to 1200 IU). All personnel were blinded to study medication.

Study investigators, site personnel, endpoint adjudicators, the Novartis clinical team, as well as the clinical research organization and Coordinating Center personnel involved in the conduct of the trial were all blinded to treatment assignments.

### Endpoints

The primary endpoint was percentage change in femoral neck BMD at year 6 relative to year 3 (baseline for this extension study). Secondary endpoints included spine and total hip BMD, biochemical bone turnover markers, and fractures (clinical, nonvertebral, clinical spine, and morphometric vertebral). Changes from pretreatment levels over 6 years (years 0 to 6) were also assessed.

### Efficacy measurements

Dual X-ray absorptiometry of the hip was performed on all participants in the core study and at years 4.5 and 6 in the extension. The value at the final core visit (year 3) was used as the extension baseline. Spine BMD was assessed in a subset of patients. Quality control and BMD scan analyses were performed centrally (Synarc, Portland, OR, USA).

Levels of serum procollagen type I N-terminal propeptide (PINP) were batch-assayed for all patients using archived serum collected at years 0, 3, 4.5, and 6. Analyses of beta C-terminal type 1 collagen telopeptide (ß-CTX) and bone-specific alkaline phosphatase (BSAP) (Synarc, Lyon, France) were done in a small subset of participants who had frozen serum from the core study.

Fractures were assessed using identical methods to those in the core study.^1^ Briefly, clinical fractures were initially identified by self-report with central adjudication from radiographic or surgical reports. Incidence of morphometric vertebral fractures was assessed by comparison of baseline (3-year) to final study radiographs using standard criteria that required both a quantitative morphometric (QM) change (20% or ≥4 mm) and change ≥1 semiquantitative (SQ) grade. Secondary analyses were performed restricting new vertebral fractures to women who met QM incident fracture criteria with SQ change ≥2 and ≥3. QM evaluation was performed on all films, and SQ was performed for confirmation if the pair met QM incident fracture criteria.^19^ Prevalent vertebral fractures at study baseline were defined by QM criteria using the modified Melton-Eastell methods.^20^, ^21^ SQ assessment of baseline radiographs was performed for all women with baseline femoral neck *T*-score >−2.5 or could have been performed in conjunction with a confirmation of a later QM incident fracture. SQ assessments were used for defining prevalent fractures only if QM was unavailable.

### Adverse events

Safety was assessed by recording all self-reported adverse events (AEs) and serious AEs (SAEs); regular monitoring of hematology, blood-chemical, and urinary values; regular measurement of vital signs; and physical examinations. AEs were coded using the Medical Dictionary for Regulatory Activities (MedDRA).

A number of specific safety evaluations were performed. All patients had serum creatinine measured 9 to 11 days after each infusion to assess renal safety. A significant increase was predefined as serum creatinine rise >0.5 mg/dL compared with preinfusion or baseline. Twelve-lead electrocardiograms (ECGs) were collected on all patients 9 to 11 days and 90 days after the year 5 infusion. Blinded, independent adjudications/expert review committees adjudicated reports of several AEs of interest: ocular; hypocalcemia; maxillofacial; avascular necrosis; delayed/nonunion of fractures; renal; arrhythmia SAEs; and underlying cause of death. After a search of MedDRA terms using lists defined by the adjudication committees or if some predefined thresholds were reached (eg, serum creatinine rise >0.5 mg/dL), the clinical sites collected medical documentation. Adjudication was performed blinded to treatment. ONJ events were adjudicated based on a definition of exposed bone for more than 6 weeks.^1^, ^22^

### Statistical analyses

The primary analysis of percentage change in femoral neck BMD from baseline (year 3) to year 6 was performed on the intent-to-treat (ITT) population in patients with complete data for this outcome. Secondary analyses were performed using most recent BMD carried forward and with multiple imputation of missing data.^23^ Analysis of variance (ANOVA) models were used with treatment and region as covariables. All testing was performed at a *p* = 0.05 significance level without adjustment for multiple testing. A study completer was defined as having the primary variable (hip BMD) available within 6 months of the 36-month study closeout target.

The years 3 to 6 bone marker analysis used analysis of covariance (ANCOVA) with treatment, region, and log (year 3) as explanatory variables using the (log_e_-transformed) postbaseline value. Between-treatment comparison of clinical fractures used proportional hazards models. Three-year incidence of clinical fractures was estimated using Kaplan–Meier methods. New morphometric vertebral fracture incidence was compared between treatments using logistic regression adjusting for number of baseline vertebral fractures (0, 1, ≥2).

Adverse event categorizations were based on previous categorization from the core study or statistical significance (*p* < 0.05) between groups in this study. All safety analyses were performed in the ITT population minus any participants (*n* = 4) who did not receive the study drug. Some categories of AEs were composed of prespecified groups of terms (Table 3). Results are based on the investigator's original report/classification.

**Table 3:** Adverse Events

	Z3P3 (*n* = 616)	Z6 (*n* = 613)	
			
AE	*n* (%)	*n* (%)	*p* Value
General adverse events			
Total subjects with any AE	552 (89.61)	552 (90.05)	0.85
Total subjects with any SAE	168 (27.27)	191 (31.16)	0.15
Total deaths	18 (2.92)	26 (4.24)	0.22
Total subjects discontinuing because of AE	11 (1.79)	14 (2.28)	0.55
Renal events	(*n* = 615)	(*n* = 612)	
Increase in serum creatinine >0.5 mg/dL^a^	4 (0.65)	18 (2.94)	0.002
Urinary protein dipstick >2+^a^	1 (0.16)	2 (0.33)	0.62
Calculated creatinine clearance <30 mL/min^b^	21 (3.60)	28 (4.86)	0.31
Most commonly occurring postdose symptoms (≤3 days)^c^			
Pyrexia	10 (1.62)	19 (3.10)	0.09
Myalgia	15 (2.44)	19 (3.10)	0.49
Influenza-like illness	5 (0.81)	8 (1.31)	0.42
Headache	16 (2.60)	20 (3.26)	0.50
Arthralgia	8 (1.3)	10 (1.6)	0.64
Any of above (after year 4 infusion)	29 (4.7)	36 (5.9)	0.38
Any of above (after year 5 infusion)	14 (2.3)	24 (3.9)	0.10
Any of above (after year 6 infusion)	7 (1.1)	14 (2.3)	0.13
Cardiovascular AEs			
Arrhythmia^d^			
Any AE	52 (8.4)	60 (9.8)	0.43
SAE	11 (1.8)	20 (3.3)	0.11
Atrial fibrillation			
Any AE	13 (2.1)	21 (3.4)	0.17
SAE	7 (1.1)	12 (2.0)	0.26
Stroke^e^			
SAE	9 (1.5)	19 (3.1)	0.06
Death from stroke^f^	0 (0.0)	4 (0.7)	0.06
Myocardial infarction			
Any AE	4 (0.6)	6 (1.0)	0.55
SAE	4 (0.6)	5 (0.8)	0.75
Hypertension^g^	93 (15.1)	48 (7.8)	0.0001
Death from cardiovascular causes^f^	3 (0.5)	8 (1.3)	0.14

AE = adverse event; SAE = serious adverse event.

a For urinary protein dipstick >2 +, an extension criterion of baseline urinary protein dipstick ≤2+ is required. All increases in creatinine clearance were temporary and resolved with additional remeasurement. All patients with increased levels 9 to 11 days after dose had resolved and could be redosed at next annual visit (years 4 and 5).

b For creatinine clearance <30 mL/min, an extension criterion of baseline creatinine clearance ≥30 mL/min is required.

c The five most common AEs reported within 3 days of infusion in the ZOL group in the core study.

d Arrthymia category includes AEs coded to MedDRA Arrthymia Higher Level Group term.

e Twenty-eight women with 35 events. Category of stroke includes selected relevant terms within the MedDRA System Organ Classification of nervous system disorders, which had been predefined for reporting strokes for regulatory submissions. Specific MedDRA terms within this set (with ≥2 events): cerebrovascular accident or cerebral infarction (total = 11; Z3P3 = 5, Z6 = 6), transient ischemic attack (TIA) (total = 9; Z3P3 = 2, Z6 = 7), cerebral hemorrhage (total = 2; Z3P3 = 0, Z6 = 2). An analysis of baseline risk factors for stroke did not suggest that the nonsignificant imbalance in strokes during the study was because of an imbalance in any baseline risk factors.

f Deaths by cause as reported by investigator before adjudication for underlying cause of death. Adjudicated causes could be assigned to 25 (57%) of 44 deaths. Among causes of death after adjudication, those the result of stroke were 1 (Z3P3) and 3 (Z6) and those the result of cardiovascular causes were 1 (Z3P3) and 0 (Z6). Cardiovascular deaths include any death in which the preferred term for the cause was in the MedDRA System Organ Classification of cardiac event.

g Preferred term of hypertension (does not include related preferred terms).

Comparisons for the incidence of safety events were performed using Fisher's exact test.

With a sample size of 1240, 5.5% standard deviation BMD, and two-sided 5% significance level, the trial had 90% power for a difference of 1.1% in femoral neck BMD.

## Results

**Fig. 1:**
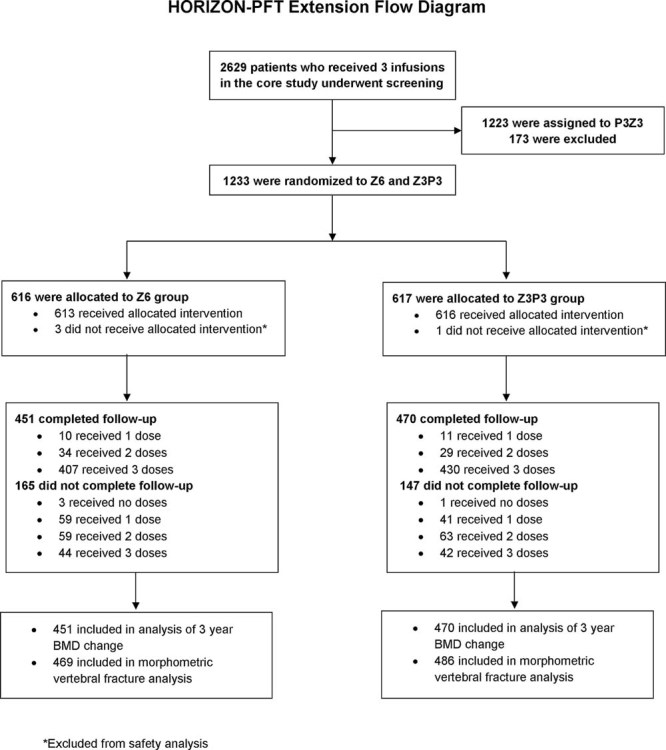
Enrollment, follow-up, and outcomes by randomized group. Complete follow-up is defined as availability of primary endpoint (year 3 to 6 change in femoral neck bone mineral density).

Baseline characteristics are shown in Table 1. On average, patients were 75.5 years old, with >50% having femoral neck BMD *T*-scores lower than −2.5 and approximately 60% with at least one vertebral fracture. Baseline characteristics were similar between treatment groups. Among the 921 (77% of survivors) women completing the study (Fig. 1), baseline characteristics were also similar in Z6 versus Z3P3 groups (Table 1). Compared with all core study patients, those in the extension were somewhat younger but had similar BMD and vertebral fracture prevalence.

**Table 1:** Baseline Characteristics of the Study Population of 1233 Subjects at the Start of Extension^a^

Variable	Z3P3 (*n* = 617)	Z6 (*n* = 616)	*p* Value
Age, years (mean ± SD)	75.5 (4.9)	75.5 (4.9)	0.89
<70, *n* (%)	78 (12.6)	67 (10.9)	
70 to 74, *n* (%)	201 (32.6)	219 (35.6)	
≥75, *n* (%)	338 (54.8)	330 (53.6)	
Mean BMI (±SD), kg/m^2^	25.6 ± 4.5	25.3 ± 4.0	0.23
Region, *n* (%)			1.0
Western Europe	226 (36.6)	222 (36.0)	
Eastern Europe	137 (22.2)	140 (22.7)	
North America/Oceania	112 (18.2)	110 (17.9)	
Latin America	115 (18.6)	116 (18.8)	
Asia	27 (4.4)	28 (4.5)	
Femoral neck *T*-score (mean ± SD)	−2.55 ± 0.57	−2.58 ± 0.55	0.29
≤−2.5, *n* (%)	325 (52.7)	353 (57.3)	0.25
>−2.5 to −1.5, *n* (%)	262 (42.5)	238 (38.6)	
>−1.5, *n* (%)	28 (4.5)	23 (3.7)	
Missing, *n* (%)	2 (0.3)	2 (0.3)	
Mean BMD (±SD), g/cm^2^			
Femoral neck	0.57 (0.06)	0.56 (0.06)	0.29
Total hip	0.69 ± 0.088	0.69 ± 0.085	0.16
Lumbar	0.82 ± 0.16	0.81 ± 0.13	0.40
Prevalent vertebral fracture, *n* (%)			0.06
0	227 (36.8)	256 (41.6)	
1	168 (27.2)	177 (28.7)	
≥2	222 (36.0)	183 (29.7)	
Prior medication use,^b^ *n* (%)			
Hormone therapy	56 (9.1)	63 (10.2)	0.50
Bisphosphonates	2 (0.3)	7 (1.1)	0.11
Calcitonin	28 (4.5)	42 (6.8)	0.09
SERMs	55 (8.9)	63 (10.2)	0.44
Other osteoporosis medication	5 (0.8)	10 (1.6)	0.21
Mean (±SD) days since last infusion in the HORIZON-PFT core study to first infusion in the extension study	394.21 ± 50.80	397.58 ± 51.86	0.25

SD = standard deviation; BMI = body mass index; BMD = bone mineral density; SERMs = selective estrogen receptor modulators.

a Plus-minus values are means ± SD.

b Reported use before HORIZON-PFT core study (before time point 0) and during core study (time point 0 to year 3).

**Fig. 2:**
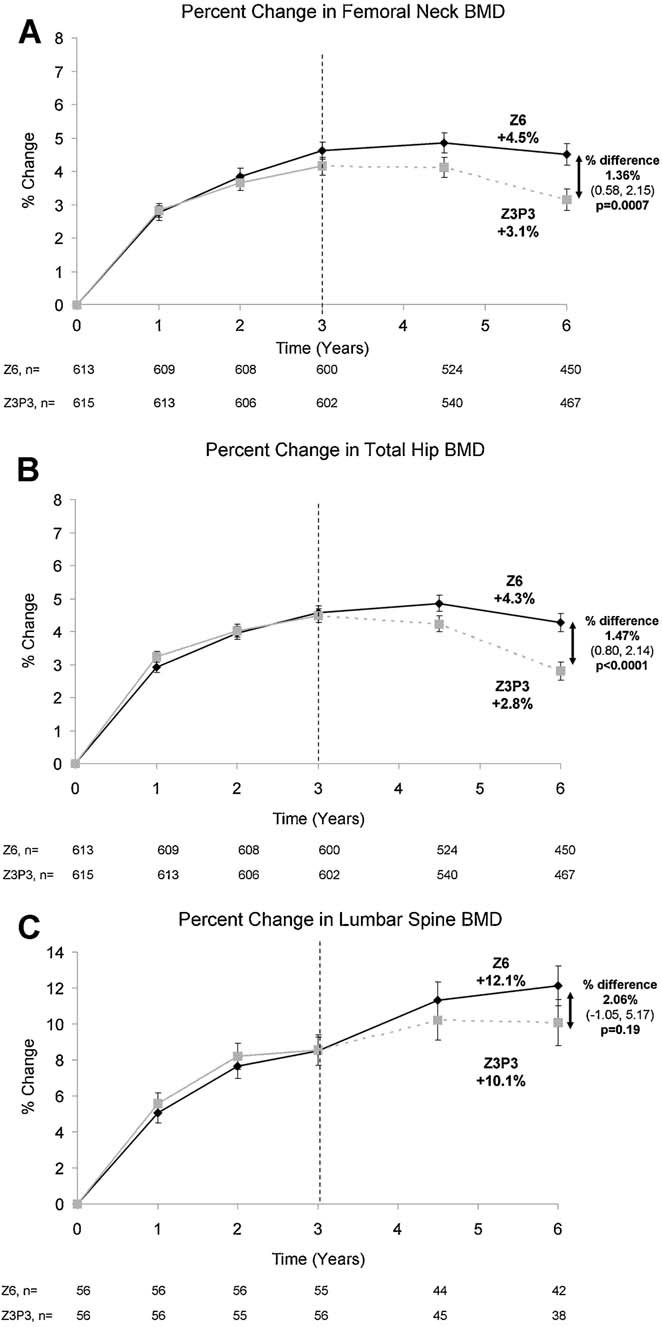
Mean changes in bone mineral density (BMD) over 6 years of treatment. The numbers at the bottom of each panel show the number of available measurements at each time point. For the core study period, only values for those continuing in the extension study are shown.

The mean change from randomization (year 3) to 6 years for femoral neck BMD (primary endpoint) was +0.24% in Z6 compared with −0.80% in Z3P3 (difference = 1.04%; *p* = 0.0009) (Table 2). Difference at the total hip was similar and also significant. The difference at the lumbar spine was slightly larger (2.03%; *p* = 0.002). Over the entire 6-year treatment/follow-up period, the gain in femoral neck and lumbar spine BMD was approximately 4.5% in the Z6 versus 3.1% in the Z3P3 group (*p* < 0.01; Fig. 2*A*) and 12.1% and 10.1%, respectively (*p* = not significant; Fig. 2*B*). Two different methods for imputing missing femoral neck BMD information (last postrandomization observation carried forward and multiple imputation) yielded similar results.

**Table 2:** Between-Treatment Comparison in Percentage Change in Bone Density and PINP at Year 4.5 and Year 6 Relative to Year 3^a^

Location	Visit	Treatment	*n*	Mean change (%)	Mean % difference (95% CI)	*p* Value
Femoral neck	Year 4.5	Z6	525	0.59	0.53 (−0.02, 1.08)	0.06
		Z3P3	544	0.06	—	—
	Year 6^b^	Z6	451	0.24	1.04 (0.43, 1.65)	<0.001
		Z3P3	470	−0.80	—	—
Total hip	Year 4.5	Z6	525	0.37	0.55 (0.18, 0.92)	0.004
		Z3P3	544	−0.18	—	—
	Year 6	Z6	451	−0.36	1.22 (0.75, 1.70)	<0.0001
		Z3P3	470	−1.58	—	—
Lumbar spine	Year 4.5	Z6	101	2.41	1.40 (0.38, 2.42)	0.01
		Z3P3	102	1.01	—	—
	Year 6	Z6	100	3.20	2.03 (0.76, 3.29)	0.002
		Z3P3	84	1.18	—	—
Distal radius	Year 4.5	Z6	100	0.45	1.32 (0.40, 2.24)	0.01
		Z3P3	99	−0.86	—	—
	Year 6	Z6	96	−0.12	0.37 (−0.71, 1.45)	0.50
		Z3P3	82	−0.49	—	—
PINP	Year 4.5	Z6	402	−23%	47%	<0.001
		Z3P3	431	+24%		
	Year 6	Z6	370	+19%	14%	0.0001
		Z3P3	395	+33%		

CI = confidence interval; PINP = serum procollagen type I N-terminal propeptide.

n is the number of patients with values at year 3 and the follow-up visit. 95% CI is calculated based on a t-distribution for BMD. The *p* value is obtained from ANOVA with treatment and region as explanatory variables.

a The 4.5-year point is 6 months after year 4 infusion: 6-year point is 12 months after year 5 infusion.

b Using the last postrandomization observation carried forward to impute missing data, the difference in femoral neck BMD change between treatment groups was 0.71% (95% CI 0.14%, 1.28%; *p* = 0.015). Using multiple imputation, the difference was 0.88% (95% CI 0.28%, 1.49%; *p* = 0.004).

**Fig. 3:**
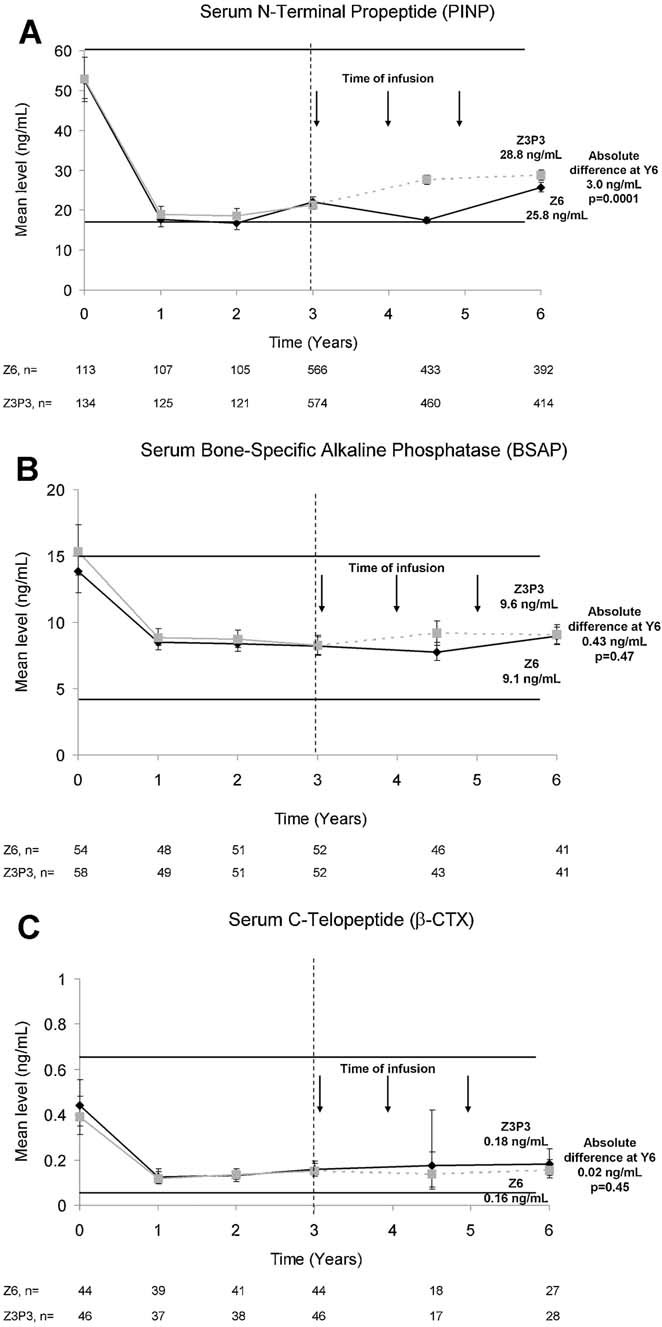
Mean changes in bone turnover markers over 6 years of treatment. The horizontal lines indicate premenopausal reference ranges,^36^ and the arrows indicate timing of infusions. The year 4.5 measurement was made 6 months after the most recent infusion, whereas the year 6 measurement was 12 months after the most recent infusion. Results represent geometric means.

During the 3 years of the extension study, mean serum PINP rose slightly in both the Z3P3 (+33%) and Z6 (+19%; difference = 14%, *p* = 0.0001) groups (Table 2). Three years after discontinuation, PINP still remained substantially below pretreatment values in Z3P3 (Fig. 3*A*). The patterns of change were similar for β-CTX and BSAP but sample sizes were too small to draw meaningful conclusions (Fig. 3*B,C*).

**Fig. 4:**
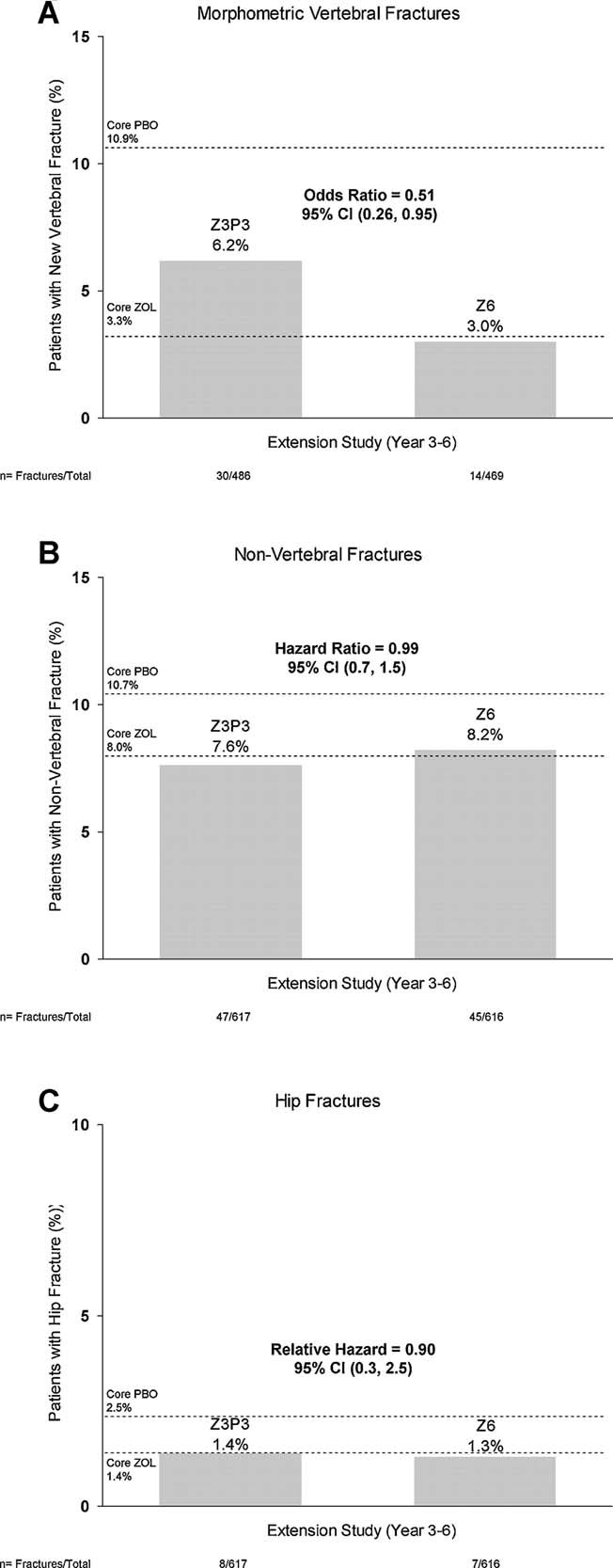
Incidence of fractures by treatment in the extension for morphometric vertebral fractures (*A*), nonvertebral fractures (*B*), and hip fractures (*C*). The dashed lines indicate the incidence in the core trial by core treatment for the corresponding fracture types. For *B* and *C*, the percentages given are the event rate from the Kaplan–Meier estimate at month 36 in the extension (bars) or core study (dashed lines).

Morphometric vertebral fracture risk was significantly lower in Z6 (*n* = 14) versus Z3P3 (*n* = 30; 3.0% versus 6.2%, odds ratio [OR] = 0.51, 95% confidence interval [CI] 0.26 to 0.95; *p* = 0.035) (Fig. 4*A*). However, there was no significant difference in fracture incidence for nonvertebral (hazard ratio [HR] = 0.99, 95% CI 0.7 to 1.5; Fig. 4*B*), clinical vertebral (HR = 1.81, 95% CI 0.53 to 6.2), hip (HR = 0.9, 95% CI 0.33 to 2.49) (Fig. 4*C*), or all clinical fractures (HR = 1.04, 95% CI 0.71 to 1.54).

Restricting morphometric vertebral fractures to those with an SQ change ≥2 yielded 11 events in Z6 versus 29 in Z3P3 (OR = 0.41, *p* = 0.01). Restricting to SQ change ≥3 yielded 8 in Z6 and 16 in Z3P3 (OR = 0.59, *p* = 0.22).

The number of women with one or more AE was similar in the two groups (Table 3), and there were no statistically significant differences in SAEs or deaths. With respect to renal effects, a significantly larger number of patients with increases in serum creatinine >0.5 mg/dL from baseline occurred in Z6 (*n* = 18) versus Z3P3 (*n* = 4; *p* = 0.002) (Table 3). The majority of these increases occurred between infusion and the 9- to 11-day postinfusion follow-up visit; all were transient and resolved with no overall impact on renal function. The mean changes in serum creatinine (µmol/L) from the extension baseline to the postinfusion follow-up visit were similar in the Z6 and Z3P3 groups. For example, after the year 3 infusion, the mean change (minimum, maximum) from baseline to the postinfusion follow-up visit was 2 (−27, 88) in Z6 and 1 (−27, 62) in Z3P3. The corresponding values following the sixth infusion were 3 (−71, 345) in Z6 and 1 (−27, 53) in Z3P3. The patient with the serum creatinine rise of 345 µmol/L had a value of 70.7 µmol/L before the sixth infusion and 424 µmol/L at the postinfusion follow-up visit. As per protocol, this parameter was remeasured 4 days later and was found to have a normal value (70.7 µmol/L) identical to the preinfusion value, suggesting a spurious measurement at the 9- to 11-day visit. Postdose symptoms were relatively uncommon and not different between treatment groups. Atrial fibrillation was slightly more common in the Z6 group, but the difference was not statistically significant. In addition, a number of other cardiovascular events, including stroke, were numerically more frequent in the Z6 group (*n* = 19) compared with the Z3P3 group (*n* = 9), but none were statistically significant. Of the 19 strokes in the Z6 group, none occurred within 30 days of infusion. After excluding the transient ischemic attack (TIA) events, there were 13 strokes in the Z6 group versus 7 in the Z3P3 group (*p* = 0.18). There was a statistically significant decrease in the Z6 group in the number of hypertension AEs reported compared with the Z3P3 group (48 versus 93, *p* = 0.0001).

There were no cases of atypical femur fractures or hip or knee avascular necrosis. There was one case meeting ONJ criteria (in Z6 group), which resolved with appropriate treatment.

## Discussion

We compared the efficacy of 6 years of continuous annual use of ZOL with discontinuation after 3 years. For the primary endpoint of femoral neck BMD (year 3 versus 6), continuous use maintained BMD gains seen after the first 3 years of ZOL, whereas discontinuation resulted in bone loss of 1.04% (compared with the Z6 group). However, BMD in both groups remained substantially above pretreatment values. Bone remodeling rates remained constant for those remaining on ZOL for 6 years, and there was only a slight increase in those who stopped therapy. For fractures, we saw 49% lower risk for morphometric vertebral fractures (*n* = 14 [3.0%] in Z6 versus *n* = 30 [6.2%] in Z3P3), but no significant difference in clinically evident vertebral fractures or nonvertebral fractures, although confidence intervals are wide. In general, fracture rates in those who continued and those who discontinued were more similar to those in the actively treated group in the core study and were lower than those seen in the placebo group in the core study. This was particularly evident for vertebral fractures. Taken together, these efficacy results show that continuing ZOL for 6 years maintains early gains in BMD and, by implication, bone strength, but discontinuation after 3 years also maintains substantial residual benefit.

In general, safety was similar in those continuing ZOL compared with those who discontinued. In the core study, there had been significant differences in acute phase response between the ZOL and placebo groups, particularly after the first infusion.^1^, ^24^ In the extension, rates of postdose symptoms were much lower than active group rates in the core study and not significantly different between randomized groups. In terms of renal effects, there were significantly more short-term rises in serum creatinine 9 to 11 days after infusion in the Z6 versus the Z3P3 group, but these short-term increases quickly resolved; there was no difference between treatment groups in mean change in creatinine clearance and there were no long-term differences in any aspect of renal function. A recent FDA warning regarding renal failure after zoledronic acid emphasized the importance of predose assessment of creatinine clearance and hydration status. It is also important that ZOL be infused over at least 15 minutes. In the original study, there were significantly greater numbers of SAE atrial fibrillation (1.3% versus 0.5%; *p* < 0.001), although no plausible mechanism or correlation to electrolyte disturbance was identified. In this extension, although there were numerically more events in the Z6 (2.0%) versus Z3P3 group (1.1%), this difference was not statistically significant (*p* = 0.26). Although SAE strokes were numerically more common in the Z6 versus Z3P3 group, that difference did not reach statistical significance (*p* = 0.06). None of the strokes occurred within 30 days of infusion and, excluding TIAs, decreased the imbalance. None of the strokes were preceded by SAE atrial fibrillation in the study, and neither our core study nor any other study of ZOL has shown a significant increase in stroke. The only statistically significant difference in this study for cardiovascular events was hypertension, for which the number of reports was significantly decreased in the Z6 versus the Z3P3 group. Given the uncertainty and inconsistency in these cardiovascular event data, we do not believe that the evidence supports any general recommendation. There was only a small increase in bone turnover in those who discontinued therapy, and levels of turnover remained substantially below pretreatment levels. The persistent decrease in turnover for at least 3 years after discontinuation suggests a residual effect, which could be beneficial for continued fracture risk reduction. There is a theoretical concern that if it is found that this degree of reduction in bone turnover has adverse effects, then these effects will be similarly prolonged. However, it should be noted that most patients have values within the premenopausal normal range, indicating that there is not a nonphysiological suppression of bone turnover in either as a result of prolonged or in those who discontinued. Thus, unexpected adverse effects from this level of turnover are extremely unlikely.

These results provide some guidance for clinicians in deciding whether to continue a patient on ZOL beyond 3 years. Changes in bone density and markers suggest statistically significant, but small differences between continuing and stopping medication after 3 years for up to 3 years. There was no difference for any type of clinical fracture, although a 49% lower risk of morphometric vertebral fractures was found in those continuing on ZOL. Incident morphometric vertebral fractures have been shown to be associated with significant pain, limited activity, disability, and increased future fracture risk.^25^–^27^ Although there was no significant decrease in clinical vertebral fractures, the confidence interval for all categories of clinical fracture were wide and therefore we cannot exclude some potential benefit. Overall, the results suggest that after 3 years of initial treatment, many patients may discontinue therapy for up to 3 years or decrease frequency of infusion. However, based on the reduction in morphometric fractures, those who are at high fracture risk, particularly vertebral fracture, may benefit from continued annual infusions. A previous study of alendronate suggested that those with existing vertebral fractures or very low BMD after initial alendronate treatment were at highest risk of new fractures and may most benefit by continuing.^8^, ^28^ Future analyses in our study will examine in detail factors that might aid clinical decision making regarding continuation: preliminary results are consistent with those from FLEX showing increased fracture risk in those who discontinued among those with very low BMD or existing vertebral fractures after an initial course of therapy.^29^ Long-term benefits must be balanced against any possible safety concerns including ONJ, atypical femur fracture, or other possible AEs. In some patients, clinicians might consider less frequent dosing, which may potentially provide similar efficacy while decreasing cumulative drug exposure,^30^, ^31^ although it has not been studied after previous annual infusions.

In comparison to our ZOL results, data on efficacy and safety of other bisphosphonates beyond 3 to 5 years varies and is limited. A similar extension study to ours was performed for alendronate.^7^, ^8^, ^32^ Patients with an average of 5 years of previous alendronate were rerandomized and continued on alendronate 5 or 10 mg/day or placebo for 5 more years. In that extension study, 3-year results were similar to ours, showing a small decline in BMD (approximately 1% to 2% at the hip and 2% to 3% at the spine), and 5-year results showed no reduction in clinical nonvertebral fractures. The alendronate extension study also showed a reduction in vertebral fractures, but only for clinical, not morphometrically defined fractures. Over the 5 years of the follow-up, there was a larger resolution of effect for bone turnover after alendronate than we showed over 3 years of follow-up after ZOL. From these data, the authors concluded that women at high risk of vertebral fractures or those with very low BMD might be best continued after 5 years of alendronate but that others could safely discontinue. In comparison to our ZOL data and those for alendronate, which show a similar residual effect, the limited data for risedronate suggest faster offset and less residual effect,^9^ and there are no long-term data for ibandronate. It is important that clinicians do not assume that the residual effects observed with ZOL and alendronate apply to other bisphosphonates.

In terms of residual effects after discontinuation, bisphosphonates are distinct from other anti-osteoporosis agents including estrogen, raloxifene, parathyroid hormone, and receptor activator of NF-κB ligand (RANKL) inhibitors, such as denosumab. For these agents, BMD rapidly decreases after discontinuation, such that BMD gains may be lost within 1 to 2 years and bone remodeling quickly returns to pretreatment levels or above.^33^–^35^ This lack of residual effect contrasts sharply with our results and those for alendronate.

Our study had several limitations. Most important, the number of patients was too small to examine uncommon clinical events, such as uncommon fracture types and AEs. Confidence intervals for fracture endpoints, as well as safety, are therefore wide, and clinical recommendations must be primarily based on BMD and bone turnover markers and surrogate endpoints for fracture. Also, there was no long-term placebo group, so we can only compare between those who received ZOL for 3 versus 6 years. Lastly, we only compared 3 additional years of ZOL to discontinuing for 3 years. Our data do not allow us to estimate whether residual effects continue longer than 3 years nor what criteria, such as change in BMD or bone turnover marker, might be used to assess if and when another course of therapy should be started.

In summary, our study showed that continuing annual ZOL over 6 years maintained BMD and reduced vertebral fracture risk. Although discontinuation after 3 years showed an increase in morphometric vertebral fractures, there was also evidence of substantial residual benefits. These residual benefits after discontinuation suggest that after 3 years, many patients may discontinue infusions for up to 3 years, decreasing costs and possible adverse effects while maintaining efficacy. However, women at high risk of fracture, particularly vertebral fracture, may benefit from continuing annual infusions.

## Disclosures

DMB: grant support from Novartis, Merck, Roche, and Amgen; consulting or advisory board fees from Eli Lilly, Amgen, Zosano, Radius, and Nycomed. IRR: consulting or advisory board fees from Merck, Amgen, and Novartis; lecture fees from Amgen, Merck, Novartis; grant support from Amgen, Novartis, Procter & Gamble, and Merck. SB: consulting or advisory board fees from Eli Lilly, Merck, Novartis, and Servier; lecture fees from Amgen, Eli Lilly, Merck, Novartis, and Servier; grant support from Amgen, Eli Lilly, Novartis, Pfizer, and Roche-GlaxoSmithKline. CB-R: full-time employee of and having an equity interest in Novartis Pharmaceutical Corporation. JAC: consulting fees from HORIZON Steering Committee and Novartis and grant support from Novartis. FC: consulting/advisory board fees from Eli Lilly, Novartis, Merck, and Amgen; lecture fees from Eli Lilly, Novartis, and Amgen; grant support from Eli Lilly and Novartis. SRC: consulting fees from Amgen, Eli Lilly, and Merck; lecture fees from Eli Lilly; grant support from Amgen. TFH: no conflicts of interest. KL participated in advisory boards of Amgen, Daiichi-Sankyo, Eli Lilly, MSD, Nycomed, Novartis, Pfizer, and Roche, and was and/or is currently involved in clinical research programs by Amgen, MSD, Novartis, Roche, and Servier. PL: consulting or advisory board fees from Amgen, Eli Lilly, Novartis, and Servier; lecture fees from Amgen, Eli Lilly, Merck, Novartis, Roche, and Servier; grant support from Amgen and Roche-GlaxoSmithKline. PCL: advisory board fees from Novartis. ZM: advisory board fees from Novartis; lecture fees from Novartis, Roche, and Sanofi-Aventis; grant support for clinical trials from Amgen, Eli Lilly, Novartis, NPS, Procter & Gamble, Roche, Sanofi-Aventis, and Servier. RM, MT, MER, and GS: full-time employees of Novartis Pharmaceuticals Corporation. RE: consulting fees from Amgen, AstraZeneca, GSK, Medtronics, Nastech, Nestle, Fonterra Brands, Novartis, Ono Pharma, Osteologix, Pfizer, Eli Lilly, Sanofi Aventis, Tethys, Unilever, Unipath, and Inverness Medical; grant support from Amgen, Eli Lilly, Warner Chilcott, and AstraZeneca.
